# Correction: Whole-Genome Analysis of a Rare Human Korean G3P[9] Rotavirus Strain Suggests a Complex Evolutionary Origin Potentially Involving Reassortment Events between Feline and Bovine Rotaviruses

**DOI:** 10.1371/journal.pone.0101404

**Published:** 2014-06-26

**Authors:** The PLOS ONE Staff

## Notice of Republication

This article was republished on June 3, 2014, to correct Figure 1, which was missing panels d-k. Please download this article again to view the correct version. The originally published, uncorrected article and the republished, corrected article are provided here for reference.

## Supporting Information

File S1 Originally published, uncorrected article.
(PDF)

File S2 Republished, corrected article.
(PDF)
