# Supplementary material for: Whole-Genome Analysis of a Rare Human Korean G3P Rotavirus Strain Suggests a Complex Evolutionary Origin Potentially Involving Reassortment Events between Feline and Bovine Rotaviruses
Source: PLoS One. 2014 May 12;9(5):e97127. doi: 10.1371/journal.pone.0097127 (PMC4018271; doi:10.1371/journal.pone.0097127)
Supplement: Table S1 — Primers used to amplify the VP7, VP4, VP6, VP1, VP2, VP3, NSP1, NSP2, NSP3, NSP4 and NSP5 gene segments described in this study. (DOC) [file pone.0097127.s001.doc]

**Table S1**. Primers used to amplify the VP7, VP4, VP6, VP1, VP2, VP3, NSP1, NSP2, NSP3, NSP4 and NSP5 gene segments described in this study

| **Gene segment** | **Primer name** | **Primer sequences (5’-3’)** | **Reference** |
| --- | --- | --- | --- |
| VP7 | Beg9 | GGCTTTAAAAGAGAGAATTTCCGTCTGG | [28] |
|  | End9 | GGTCACATCATACAATTCTAATCTAAG |  |
| VP4 | VP4-1-1F | GGCTATAAAATGGCTTCTTT | In this study |
|  | VP4-1-1180R | CAGGCAACGCAAAACTGTAA | In this study |
|  | VP4-2-885F | CAAATCAGGTGGCCTAGGTT | In this study |
|  | VP4-2-1772R | AACGTAATAGCGCCCACTGA | In this study |
|  | VP4-3-1574R | CGCTACCGCTTGATATGTTC | In this study |
|  | VP4-3-2359R | GGTCACATCTTAAAATAGACAG | In this study |
| VP6 | GVAP6-C1 (+) | GGCTTTAAAACGAAGTCTTCAAC | [29] |
|  | VP6-1-827R | TTGCCCATTCAACAGAAACTC | In this study |
|  | VP6-1-556F | GTACAATGTGGTTGAATGCAG | In this study |
|  | VP6-1-1356R | GGTCACATCCTCTCACTATAC | In this study |
| VP1 | GEN-VP1F | GGC TATTAA AGC TAT ACA ATG GG | [30] |
|  | VP1-1-77R | CAAGAATTGACATTGGTGACG | In this study |
|  | VP1-2-627F | AGTAACATGGGCAAACTCTTCA | In this study |
|  | VP1-2-1613R | TGTGTGTTATGCTGGGATGAA | In this study |
|  | VP1-3-1375F | CAT ATCTAA ACCCAT AATTAT TCC | [31] |
|  | VP1-3-2260R | ATG ACCCAG TAA TAGCGACC | In this study |
|  | VP1-4-2130F | TAC ACA ATG GGATCA AGCAG | In this study |
|  | VP1-4-3300R | TCA CATCTA AGC GCTCTA ATC | In this study |
| VP2 | GEN-VP2F | GGCTATTAA AGGCTC AATGG | [30] |
|  | VP2-1-612R | ACTTTACCAGCGTCCCTTGA | In this study |
|  | VP2-2-418F | TAGGGCGAATGGTGA AAA AG | In this study |
|  | VP2-2-1614R | ATTCCACGCTGA ATTGACCT | In this study |
|  | VP2-3-1418F | GTAGCGAATTGGCTGCATTT | In this study |
|  | VP2-3-2253R | TGTGCATAATCACCTGTCCTC | In this study |
|  | VP2-4-2040F | AGGTGAGACGATTGGACATC | In this study |
|  | VP2-4-2683R | ATATCTCCACAGTGGGGTTG | In this study |
| VP3 | VP3-1-1F | GGCTATTAA AGCAGTACTAGTAG | [31] |
|  | VP3-1-913R | AGCTGAACCAAGCATGTATATTA |  |
|  | VP3-2-723F | TAAAGCAGTTTTCAAATGGACC | In this study |
|  | VP3-2-1780R | TCATATTTGATGAAACGTCG | In this study |
|  | VP3-3-1550F | AATTTTAAAAATATTTATGATTGGAC | [31] |
|  | VP3-3-R | GGTCACATCATGACTAGTGTG |  |
| NSP1 | NSP1-F | GGCTTTTTTTATGAAAAGTCTTGTG |  |
|  | NSP1-R | GGTTCACATTTTATGCTGCCTAG |  |
| NSP2 | GEN-NSP2F | GGCTTTTAAAGCGTCTCAG | [12] |
|  | GEN-NSP2R | GGTCACATAAGCGCTTTC |  |
| NSP3 | GEN-NSP3F | GGCTTTTAATCTTTTCAGTG |  |
|  | GEN-NSP3R | ACATAACGCCCCTATAGC |  |
| NSP4 | GEN_NSP4F | GGCTTTTAAAAGTTCTGTTC |  |
|  | GEN_NSP4R | GGTCACACTAAGACCATTCC |  |
| NSP5 | GEN-NSP5F | GGCTTTTAA AGCGCTACAG |  |
|  | GEN-NSP5R | GGTCACAAAACGGGAGT |  |
