# Supplementary material for: Whole-Genome Analysis of a Rare Human Korean G3P Rotavirus Strain Suggests a Complex Evolutionary Origin Potentially Involving Reassortment Events between Feline and Bovine Rotaviruses
Source: PLoS One. 2014 May 12;9(5):e97127. doi: 10.1371/journal.pone.0097127 (PMC4018271; doi:10.1371/journal.pone.0097127)
Supplement: Table S2 — Related origin of the CAU12-2-51 rotavirus strain to reference strains used in this study. (DOC) [file pone.0097127.s002.doc]

**Table S2**. Related origin of the CAU12-2-51 rotavirus strain to reference strains used in this study

| **Gene segment** | **Strain name** | **Origin** | **Reference** |
| --- | --- | --- | --- |
| **DS-1-like genogroup** | | | |
| VP6 | RVA/Human-wt/ITA/**PAH136**/1996/G3P[9] | human/bovine-like rotavirus | [40] |
|  | RVA/Human-wt/HUN/**Hun5**/1997/G6P[14] | human/bovine-like rotavirus | [37] |
|  | RVA/Human-wt/JPN/**KF17**/2010/G6P[9] | human/bovine-like rotavirus | [39] |
|  | RVA/Cat-wt/ITA/**BA222**/2005/G3P[9] | feline/bovine-like rotavirus | [19] |
|  | RVA/Human-wt/RUS/**Nov10-507**/2010/G3P[9] | unidentified | - |
|  | RVA/Human-wt/RUS/**RUS-Nov06-K2**/2006/G3P[9] | unidentified | - |
| VP1 | RVA/Human-wt/ITA/**PAH136**/1996/G3P[9] | human/bovine-like rotavirus | [40] |
|  | RVA/Human-wt/ITA/**PAI11**/1996/G2P[4] | human/bovine-like rotavirus | [44] |
|  | RVA/Human-wt/HUN/**Hun5**/1997/G6P[14] | human/bovine-like rotavirus | [37] |
|  | RVA/Human-wt/HUN/**BP1879**/2003/G6P[14] | human/bovine-like rotavirus | [38] |
|  | RVA/Human-wt/JPN/**KF17**/2010/G6P[9] | human/bovine-like rotavirus | [39] |
|  | RVA/Human-tc/PHL/**L26**/1987/G12P[4] | human/bovine-like rotavirus | [41] |
| VP2 | RVA/Human-wt/ITA/**PAH136**/1996/G3P[9] | human/bovine-like rotavirus | [40] |
|  | RVA/Human-wt/HUN/**Hun5**/1997/G6P[14] | human/bovine-like rotavirus | [37] |
|  | RVA/Antelope-wt/ZAF/**RC-18-08**/G6P[14] | human/bovine-like rotavirus | [37] |
|  | RVA/Cat-wt/ITA/**BA222**/2005/G3P[9] | feline/bovine-like rotavirus | [19] |
|  | RVA/Cow-tc/USA/**NCDV**/1967/G6P6[1] | bovine rotavirus | - |
|  | RVA/Cow-tc/USA/**WC3**/1981/G6P[5] | bovine rotavirus | [12] |
|  | RVA/Cow-tc/FRA/**RF**/1982/G6P[1] | bovine rotavirus | - |
|  | RVA/Cow-wt/ZAF/**1603**/2007/G6P[5] | bovine rotavirus | [45] |
|  | RVA/Cow-wt/ZAF/**1605**/2007/G6P[5] | bovine rotavirus | [45] |
| VP3 | RVA/Human-tc/ITA/**PA169**/1988/G6P[14] | human/bovine-like rotavirus | [12] |
|  | RVA/Human-wt/ITA/**PAI58**/1996/G3P[9] | human/bovine-like rotavirus | [40] |
|  | RVA/Human-wt/ITA/**PAH136**/1996/G3P[9] | human/bovine-like rotavirus | [40] |
|  | RVA/Human-wt/BEL/**B1711**/2002/G6P[6] | human/bovine-like rotavirus | [43] |
|  | RVA/Human-wt/JPN/**KF17**/2010/G6P[9] | human/bovine-like rotavirus | [39] |
|  | RVA/Cat-wt/ITA/**BA222**/2005/G3P[9] | feline/bovine-like rotavirus | [19] |
|  | RVA/Cow-tc/FRA/**RF**/1982/G6P[1] | bovine rotavirus | - |
|  | RVA/Cow-tc/USA/**NCDV**/1967/G6P6[1] | bovine rotavirus | - |
|  | RVA/Cow-tc/USA/**WC3**/1981/G6P[5] | bovine rotavirus | [12] |
| NSP2 | RVA/Human-wt/ITA/**PAI58**/1996/G3P[9] | human/bovine-like rotavirus | [40] |
|  | RVA/Human-wt/JPN/**KF17**/2010/G6P[9] | human/bovine-like rotavirus | [39] |
|  | RVA/Human-tc/ITA/**PA260-97**/1997/G3P[3] | human/bovine-like rotavirus | [46] |
|  | RVA/Dog-tc/ITA/**RV198-95**/1995/G3P[3] | canine/bovine-like rotavirus | [46] |
|  | RVA/Dog-tc/ITA/**RV52-96**/1996/G3P[3] | canine/bovine-like rotavirus | [46] |
|  | RVA/Human-wt/RUS/**O211**/2007/G3P[9] | unidentified | - |
|  | RVA/Guanaco-wt/ARG/**Chubut**/1999/G8P[14] | guanaco/bovine-like rotavirus | [37] |
|  | RVA/Cow-tc/USA/**NCDV**/1971/G6P[1] | bovine rotavirus | - |
|  | RVA/Cow-tc/USA/**WC3**/1981/G6P[5] | bovine rotavirus | [12] |
| **AU-1-like genogroup** | | | |
| VP7 | RVA/Human-wt/THA/CU365-KK/08/2008/G3P[9] | human rotavirus | - |
|  | RVA/Human-tc/CHN/L621/2006/G3P[9] | human rotavirus | [51] |
|  | RVA/Human-wt/THA/CMH120/04/2004/G3P[9] | human rotavirus | [20] |
|  | RVA/Human-wt/THA/CMH134/04/2004/G3P[9] | human rotavirus | [20] |
| VP4 | RVA/Human-wt/JPN/KF17/2010/G6P[9] | human/feline-like rotavirus | [39] |
|  | RVA/Cat-tc/AUS/Cat2/1984/G3P[9] | feline rotavirus | [18] |
|  | RVA/Cat-wt/JPN/FRV1/1985/G3P3[9] | feline rotavirus | - |
|  | RVA/Cat-wt/ITA/BA222/2005/G3P[9] | feline rotavirus | [19] |
| NSP1 | RVA/Human-wt/ITA/**PAH136**/1996/G3P[9] | human/feline-like rotavirus | [40] |
|  | RVA/Human-wt/ITA/**PAI58**/1996/G3P[9] | human/feline-like rotavirus | [40] |
|  | RVA/Human-wt/JPN/**KF17**/2010/G6P[9] | human/feline-like rotavirus | [39] |
|  | RVA/Guanaco-wt/ARG/**Chubut**/1999/G8P[14] | guanaco/feline-like rotavirus | [37] |
|  | RVA/Cat-tc/AUS/**Cat2**/1984/G3P[9] | feline rotavirus | [18] |
|  | RVA/Cat-wt/ITA/**BA222**/2005/G3P[9] | feline rotavirus | [19] |
| NSP3 | RVA/Human-tc/JPN/**K8**/1977/G1P[9] | human/feline-like rotavirus | [47] |
|  | RVA/Human-wt/JPN/**KF17**/2010/G6P[9] | human/feline-like rotavirus | [39] |
|  | RVA/Cat-wt/ITA/**BA222**/2005/G3P[9] | feline rotavirus | [19] |
|  | RVA/Human-wt/CHN/**E2451**/2011/G3P[9] | unidentified | - |
| NSP4 | RVA/Human-wt/BRA/**RV10109**/2008/G3P[9] | human/feline-like rotavirus | [48] |
|  | RVA/Human-wt/JPN/**KF17**/2010/G6P[9] | human/feline-like rotavirus | [39] |
|  | RVA/Human-wt/RUS/**N.N.12871**/XXXX/G3P[9] | unidentified | - |
| NSP5 | RVA/Human-wt/ITA/**PAH136**/1996/G3P[9] | human/feline-like rotavirus | [40] |
|  | RVA/Human-wt/JPN/**KF17**/2010/G6P[9] | human/feline-like rotavirus | [39] |
|  | RVA/giraffe-wt/IRL/**UCD**/2007/G10P[11] | unidentified | - |
|  | RVA/Cat-wt/ITA/**BA222**/2005/G3P[9] | feline rotavirus | [19] |
