# Supplementary material for: Correction: Whole-Genome Analysis of a Rare Human Korean G3P[9] Rotavirus Strain Suggests a Complex Evolutionary Origin Potentially Involving Reassortment Events between Feline and Bovine Rotaviruses
Source: PLoS One. 2014 Jun 26;9(6):e101404. doi: 10.1371/journal.pone.0101404 (PMC4072792; doi:10.1371/journal.pone.0101404)
Supplement: File S2 — Republished, corrected article. (PDF) [file pone.0101404.s002.pdf]

OPEN ACCESS Freely available online

PLOS | ONE

Whole-Genome Analysis of a Rare Human Korean G3P[9] Rotavirus Strain Suggests a Complex Evolutionary Origin Potentially Involving Reassortment Events between Feline and Bovine Rotaviruses

Sunyoung Jeong[1], Van Thai Than[1], Inseok Lim[2], Wonyong Kim[1]*

1 Department of Microbiology, Chung-Ang University College of Medicine, Seoul, South Korea, 2 Department of Pediatrics, Chung-Ang University College of Medicine, Seoul, South Korea

Abstract

A rare human rotavirus, G3P[9] strain RVA/Human-tc/KOR/CAU12-2-51/2013/G3P[9], was isolated from the stool of a 9-year-old female hospitalized with acute watery diarrhea in August 2012 in South Korea using a cell culture system, and its genome was analyzed. The complete genomic constellation of the CAU12-2-51 strain revealed a novel genotype constellation for human rotavirus, G3-P[9]-I2-R2-C2-M2-A3-N2-T3-E3-H3. Phylogenetic analysis revealed that the CAU12-2-51 strain originated from feline- and bovine-like reassortment strains. The genes encoding VP4, VP7, NSP1, NSP3, NSP4, and NSP5 were related to human/feline-like and feline rotavirus strains, whereas the remaining five genes encoding VP1, VP2, VP3, VP6, and NSP2 were related to the human/bovine-like and bovine rotavirus strains. This novel strain was identified for the first time, providing evidence of feline/bovine-to-human transmission of rotavirus. The data presented herein provide information regarding rotavirus diversity and evolution.

Citation: Jeong S, Than VT, Lim I, Kim W (2014) Whole-Genome Analysis of a Rare Human Korean G3P[9] Rotavirus Strain Suggests a Complex Evolutionary Origin Potentially Involving Reassortment Events between Feline and Bovine Rotaviruses. PLoS ONE 9(5): e97127. doi:10.1371/journal.pone.0097127

Editor: Karol Sestak, Tulane University, United States of America

Received February 21, 2014; **Accepted** April 14, 2014; **Published** May 12, 2014

Copyright: © 2014 Jeong et al. This is an open-access article distributed under the terms of the Creative Commons Attribution License, which permits unrestricted use, distribution, and reproduction in any medium, provided the original author and source are credited.

Funding: This research was supported by the Basic Science Research Program through the National Research Foundation of Korea (NRF) (2013R1A1A2A10012148) and the Brain Korea 21 PLUS Program funded by the Ministry of Education. The funders had no role in study design, data collection and analysis, decision to publish, or preparation of the manuscript.

Competing Interests: The authors have declared that no competing interests exist.

* E-mail: kimwy@cau.ac.kr

Introduction

Rotaviruses are the major cause of acute gastroenteritis in young children worldwide. Each year, these viruses are associated with deaths of approximately 453,000 children younger than 5 years of age, most of whom reside in low-income countries in Africa and Asia, where access to potable water, sanitation, and medical care is often limited [1]. *Rotavirus*, a member of the family Reoviridae, is classified into eight species/groups (A–H) [2]. Its genome is composed of 11 gene segments of double-stranded RNA that encode six structural viral proteins (VP1–VP4, VP6, and VP7) and six nonstructural proteins (NSP1–NSP6) [3].

The outer capsid of rotaviruses contains two proteins, the glycoprotein VP7 and the protease-sensitive VP4, which are used to discriminate G and P genotypes, respectively. VP7 and VP4 play major roles in eliciting the production of neutralizing antibodies in the host immune response to rotavirus infection [4]. At least 27 G and 37 P genotypes have been reported to date [5,6]. Of these genotypes, epidemiological studies have shown that five G (G1–G4 and G9) and three P (P[4], P[6], and P[8]) genotypes are the most prevalent genotypes associated with global human rotavirus infections [7]. The most common G/P genotypes are G1P[8], G2P[4], G3P[8], G4P[8], and G9P[8] [7–9], and emergence of the G12 genotype in combination with P[8] or P[6] was recently reported [10]. A rare G/P combination, G3P[9], was first detected in humans in Japan in 1982 (AU-1 strain) [11,12,13]. Such G3P[9] strain was detected in a cat in Australia in 1984 (Cat2 strain) and has since been commonly detected in cats [14–17]. Recently, reassortants of feline/human or feline/canine G3P[9] rotaviruses have been reported in humans, which has serious implications for future strategies aimed at rotavirus vaccine development [18–21].

In South Korea, rotavirus is the most common viral agent causing acute diarrhea and gastroenteritis in young children, especially during the winter season. The distributions of the G and P genotypes show temporal and geographical fluctuations [22]. The major human G types are G1–G4 and G9, combined with the P types P[4], P[6], and P[8] [23,24]. Furthermore, unusual G and P combination genotypes, including G3P[3], G3P[9], G3P[10], G8P[8], G11P[4], G11P[25], and G12P[6], have been isolated from South Korea [25]. From 2003 to 2005, the G3 genotypes were predominant [26]. Although the P[9] genotype has been detected in some rotavirus surveillance studies in South Korea, no significant emergence of P[9] genotypes has been observed in the Korean population [22]; however, no studies have specifically focused on this genotype. Therefore, further analysis of the circulating Korean genotype P[9] is needed to understand its origin, genetic variation, and potential impact on the human population.

Recently, a complete genome classification based on a nucleotide sequence analysis was defined according to established

nucleotide percent cutoff values for rotavirus [27]. According to this classification, the acronyms Gx-P[x]-Ix-Rx-Cx-Mx-Ax-Nx-Tx-Ex-Hx are used to classify VP7-VP4-VP6-VP1-VP2-VP3-NSP1-NSP2-NSP3-NSP4-NSP5/6-encoding gene segments, respectively [11,12]. In the current study, the G3P[9] rotavirus strain, RVA/Human-wt/KOR/CAU12-2-51/2012/G3P[9] (CAU12-2-51), was isolated using a cell culture system. The complete genomic sequence of this G3P[9] virus was determined after two cell culture passages and analyzed in order to provide a better understanding of the origin and genetic composition of this viral strain.

Materials and Methods

Ethics statement

The stool samples used in this study were collected and analyzed according to methods detailed in our study protocol (number #2011-10-06), which was approved by the Human Subjects Institutional Review Board (IRB) of Chung-Ang University College of Medicine, Seoul, Korea. Written informed consent was obtained from all participants in this study. For the children enrolled in this study, written informed consent was obtained from their parents. The provision of informed consent also included permission to use the data for future research purposes under conditions of anonymity.

Patient history and rotavirus identification

The stool specimen was collected from a 9-year-old girl who was hospitalized for severe gastroenteritis in August 2012 at Chung-Ang University Hospital, Seoul, South Korea. The patient was hospitalized for three days with episodes of watery diarrhea (three times/day), pain in the right upper quadrant of the abdomen, and vomiting (nonprojectile and nonbilious, containing consumed food). At that time, the patient had a hemoglobin level of 13.8 g/μL, a white blood cell (WBC) count of 8,058 WBCs/μL, and a platelet count of 236,000 cells/μL. The stool WBC count was 0–1/high-power field. The patient had not received any prior rotavirus vaccines and had not had any contact with animals (e.g., cats, dogs, or cows) at least 30 days before hospitalization. Human group A rotavirus strain was then detected from the stool sample by both enzyme-linked immunosorbent assay (ELISA) and reverse transcription-polymerase chain reaction (RT-PCR). This strain was named CAU12-2-51. The stool culture produced no growth for pathogenic bacteria, such as *Salmonella* and *Shigella*, which commonly cause diarrhea.

Rotavirus isolation

MA104 cells were acquired from the Korean Cell Line Bank (Seoul, South Korea) and grown in minimum essential medium-alpha (MEM-α; Gibco BRL, Grand Island, NY, USA) containing 5% fetal bovine serum (FBS; Gibco BRL) and 0.1% gentamicin (Gentamicin Reagent Solution; Gibco BRL) at 37°C in the presence of 5% CO_2. A G3P[9]-positive stool sample was diluted 10-fold in phosphate-buffered saline (PBS; pH 7.4) and clarified by centrifugation at 10,000×*g* for 10 min. The supernatant was filtered using a 0.45-μm sterile syringe filter (Corning Costar, Corning, NY, USA), treated with 10 μg/mL Trypsin 250 (Becton Dickinson, Sparks, MD, USA) for 30 min at 37°C. The supernatant was then inoculated onto MA104 cells in glass tubes and incubated with MEM-α in the presence of trypsin (5 μg/mL), with constant rotation during incubation. Cells were harvested 5–7 days after infection and subsequently passaged with MA104 cells until a cytopathic effect (CPE) was achieved. The rotavirus remaining in the culture fluid after the final passage was examined by immunofluorescence and RT-PCR.

RNA extraction and RT-PCR

Viral double-stranded RNA was extracted using TRIzol reagent (Gibco BRL Life Technologies) according to the manufacturer's instructions. Extracted RNA was resuspended in RNase-free water and stored at −80°C until use. The extracted RNA was then subjected to one-step RT-PCR using primer sets for the full amplification of all 11 gene segments (Table S1) [12,28,29,30,31].

Nucleotide sequencing and sequence analysis

All amplified PCR products were purified using a QIAquick PCR Purification Kit (Qiagen, Westburg, Germany) and then sequenced using a BigDye Terminator Cycle Sequencing Kit and an automated DNA sequencer (Model 3730; Applied Biosystems, Foster City, CA, USA). The resulting open reading frame (ORF) of the genes and deduced amino acid (aa) sequences were aligned using the CLUSTAL_X 1.81 program [28] and Lasergene software (DNASTAR, Madison, WI, USA). Data were compared with the corresponding rotavirus sequences from the National Center for Biotechnology Information GenBank database. The nucleotide (nt) sequences obtained in this study were deposited in GenBank under the accession numbers KJ187594–KJ187604.

Phylogenetic analysis

The nt sequences of the 11 segments of the CAU12-2-51 strain were compared with representative rotavirus sequences available from the GenBank database. Phylogenetic trees were constructed using neighbor-joining algorithms [25] from the PHYLIP suite [29] and the Kimura two-parameter model using MEGA5.03 software [30,31]. Evolutionary distances for the neighbor-joining analysis were based on the model described by Jukes et al. [32]. Tree topology was evaluated using the bootstrap resampling method with 1000 replicates of the neighbor-joining dataset with the SEQBOOT and CONSENSE programs from the PHYLIP suite.

Results

Rotavirus genotyping and isolation

The *VP7* and *VP4* nt sequences of the CAU12-2-51 strain obtained from the first-round PCR products were identical to the sequences of G3 and P[9] genotypes available in the GenBank database. CPEs were observed after two culture passages when MA104 cells exhibited obscure borders, fusion, rounding, and detachment from the walls of the tubes (data not shown).

Gene sequence comparisons and phylogenetic analysis

The complete full-length nt and deduced aa sequences of the CAU12-2-51 strain were obtained. For reference, only the strains that contained complete or at least 50% sequence availability of ORFs, as suggested by Matthijnssens et al. [27], were used.

Full genome-based classification. The CAU12-2-51 strain was found to possess the G3-P[9]-I2-R2-C2-M2-A3-N2-T3-E3-H3 genotype constellation for the VP7-VP4-VP6-VP1-VP2-VP3-VP4-NSP1-NSP2-NSP3-NSP4-NSP5/6 genes, respectively (Table 1, Figure 1). The *VP7*, *VP4*, *NSP1*, *NSP3*, *NSP4*, and *NSP5* genes were related to the human/feline AU-1-like genotype and Australian feline strains. The remaining five genes, namely, *VP6*, *VP1–VP3*, and *NSP2*, were related to the human/bovine DS-1-like and bovine-like G6 human strains.

***VP4* gene.** Phylogenetic analysis of the P[9] genotypes revealed five distinct clusters, in which the VP4/P[9] genotype of the prototype strain AU-1 was assigned to cluster a. Phylogenetic

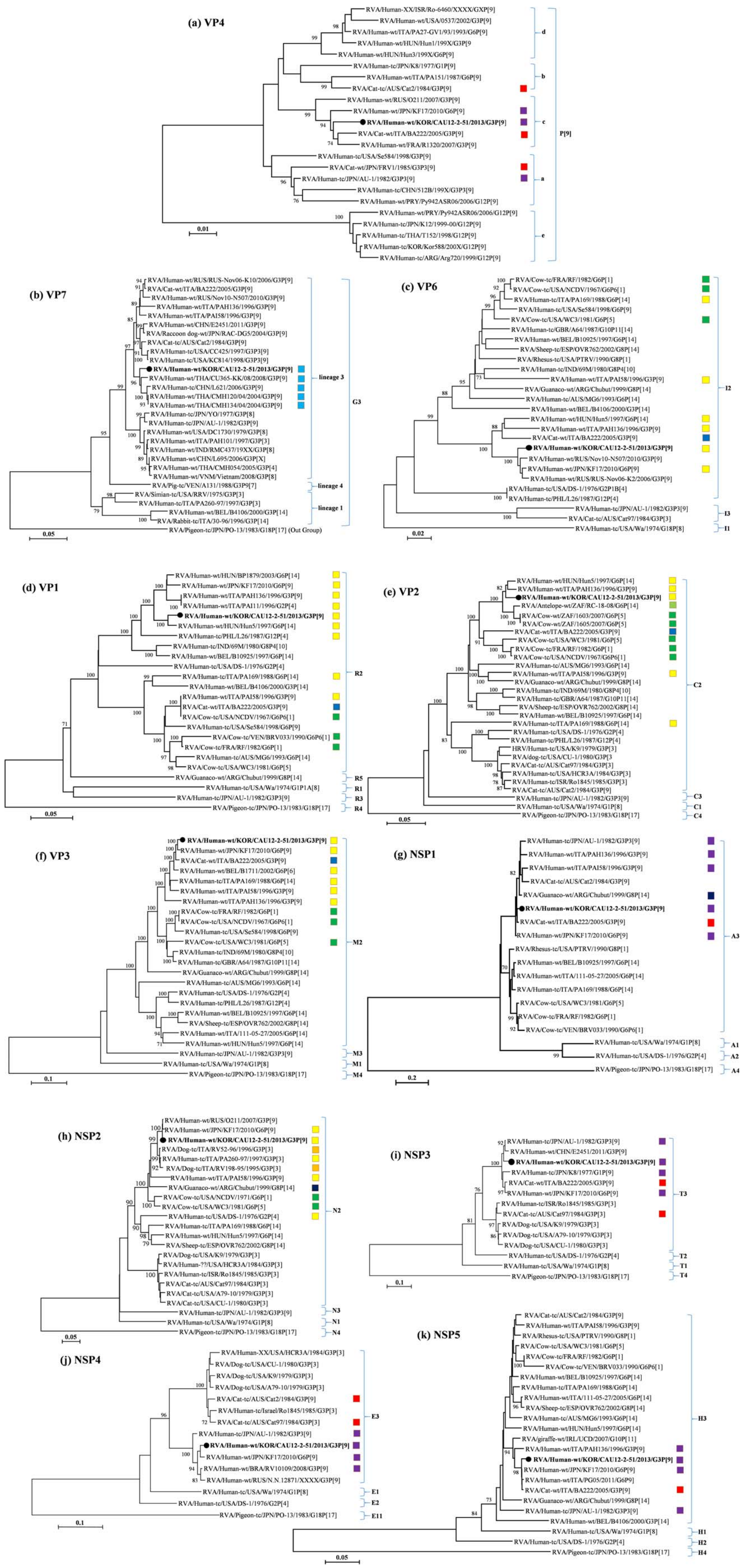
(a) VP4
(b) VP7
(c) VP6
(d) VP1
(e) VP2
(f) VP3
(g) NSP1
(h) NSP2
(i) NSP3
(j) NSP4
(k) NSP5

Figure 1. Phylogeny of the 11 genomic segments of the CAU12-2-51 strain. The different genotypes within each segment are indicated. Numbers at the nodes indicate the level of bootstrap support (%) based on neighbor-joining analysis of 1000 resampled datasets. Only values above 70% are provided. The CAU12-2-51 strain is marked in bold type and indicated by filled circles. Color coded boxes are used to differentiate strains of origin: human (light blue), bovine (green), feline (red), human/bovine-like (yellow), human/feline-like (purple), feline/bovine-like (blue), canine/bovine-like (orange), guanaco/feline-like (dark blue), and antelope/bovine-like (light green). Genotype origins are designated as follows: G, glycosylated; P, protease sensitive; I, intermediate capsid shell; R, RNA-dependent RNA polymerase; C, core shell protein; M, methyltransferase; A, interferon antagonist; N, NTPase; T, translation enhancer; E, enterotoxin; H, phosphoprotein.
doi:10.1371/journal.pone.0097127.g001

analysis showed that the VP4/P[9] genotype of the CAU12-2-51 strain was classified into cluster c, together with the recently identified human and feline P[9] genotypes, including the feline P[9] strain isolated in Italy (BA222) and human/feline-like strains isolated in France (R1320), Japan (KF17), and Russia (O211) (Figure 1a). Interestingly, VP4/P[9] of the CAU12-2-51 strain showed the highest nt and aa sequence identities to the G3P[9] feline/human reassortant BA222 strain (97.9% and 99.2%, respectively). On the other hand, VP4/P[9] of the CAU12-2-51 strain showed lower sequence identity to VP4/P[9] of the prototype strain AU-1 (95.4% at the nt level and 97.3% at the aa level).

***VP7* gene.** Phylogenetic analysis revealed that the VP7/G3 genotype of the CAU12-2-51 strain was clustered into lineage 3, together with recent human G3P[9] strains isolated from Thailand (CU365-KK, CMH120, and CMH134) and China (L621), showing the highest nt and aa sequence identities (>96% and > 97.5%, respectively; Figure 1b). In contrast, the VP7/G3 genotype of the CAU12-2-51 strain showed lower nt and aa sequence identities (90.2% and 94.1%, respectively) to the G3 prototype strain AU-1 (Table 2).

***VP1*, *VP2*, *VP3*, *VP6*, and *NSP2*.** Matthijnssens et al. demonstrated that human rotavirus strains belong to the DS-1-like genogroup (G2-P[4]-I2-R2-C2-M2-A2-N2-T2-E2-H2), sharing the VP6, VP1, VP2, VP3, NSP2, and NSP4 genotypes (I2-R2-C2-M2-N2-E2) with those of bovine rotavirus strains [12]. In this study, genotypes of VP6-VP1-VP2-VP3-NSP2 genes of the CAU12-2-51 strain were classified within the DS-1-like genogroup (I2-R2-C2-M2-N2), commonly seen in human/bovine-like and bovine G6 rotavirus strains (Figure 1c–f and 1h). However, the sequence identities of these genes to DS-1 were relatively low, i.e., just above the cutoff values, showing nt (aa) sequence identities of 86.2% (98.4%), 86.3% (97.4%), 84.8% (96.9%), 83.6% (90.9%), and 86.2% (93.6%) for *VP6*, *VP1*, *VP2*, *VP3*, and *NSP2* genes, respectively (Table 2). The *VP6*, *VP1*, *VP2*, *VP3*, and *NSP2* genes of the CAU12-2-51 strain showed the highest nt (aa) sequence identities to sequences of human/bovine-like rotavirus strains isolated in Hungary (strain Hun5 for *VP1* and *VP2*), Japan (strain KF17 for *VP3*), Russia (strain RUS-Nov-K2 for *VP6*), and Russia (strain O211 for *NSP2*) at 98.5% (99.7%), 97.4% (99.1%), 97.2% (99.4%), 97.8% (97.2%), and 99.4% (99.6%), respectively (Table 2). The *VP6* and *VP1* gene segments of the CAU12-2-51 strain clustered closely with strains originating from human/bovine-like rotavirus strains (BP1879, Hun5, KF17, PAH136, PAI11-96, Nov10-N507, and RUS-Nov06-K2), a feline/bovine-like G3P[9] strain (BA222), and a human/bovine-porcine like G12P[4] strain (L26; Figure 1c, 1d, and Table S2) [19,33–37]. In contrast, *VP2*, *VP3*, and *NSP2* gene segments were found to cluster together with most of the known rotavirus strains originating from human/bovine-like G6P[14] rotaviruses (B1711, Hun5, PAH136, PAI58, and KF17), a feline/bovine-like G3P[9] rotavirus (BA222), and bovine rotaviruses (NCDV, RF, WC3, 1063, and 1065; Figure 1e, 1f, 1h, and Table S2) [12,19,37,39,40,42,43,44,45,46].

***NSP1*, *NSP3*, *NSP4*, and *NSP5*.** Based on RNA-RNA hybridization, the human AU-1-like genogroup (G3-P[9]-I3-R3-C3-M3-A3-N3-T3-E3-H3) is believed to have a close evolutionary relationship with canine and feline rotavirus strains [12]. Our study indicated that the *NSP1*, *NSP3*, *NSP4*, and *NSP5* genes of the CAU12-2-51 strain were classified with the genotypes of strains with AU-1-like genotypes, showing nt (aa) sequence identities of 93.1% (93.1%), 96.9% (97.7%), 96.2% (97.1%), and 93.1% (92.9%), respectively (Figure 1g and 1i–k). On the other hand, the *NSP1*, *NSP4*, and *NSP5* genes of the CAU12-2-51 strain showed the highest sequence identities with the G6P[9] genotype of the human/feline-like KF17 strain, with nt (aa) identities of 97.2% (96.7%), 97.7% (97.1%), and 98.8% (97.9%), respectively. Additionally, the *NSP3* gene showed the highest nt (aa) sequence identity to the AU-1 strain at 96.9% (97.7%) (Table 2). Phylogenetic analysis indicated that the NSP1-NSP3-NSP4-NSP5 gene segments found in A3-T3-E3-H3 genotypes clustered closely with the strains originating from human/feline-like rotaviruses (K8, KF17, PAH136, PAI58, and RV10109), a guanaco/feline-like rotavirus (Chubut), and feline rotaviruses (Cat2 and BA222; Figure 1g, 1i–1k, and Table S2) [18,19,33,35,36,40,41].

Discussion

Rotavirus infection causes acute diarrhea and is the most common cause of gastroenteritis affecting infants and young children in South Korea. The human rotavirus G1–G4 genotypes account for almost all rotavirus infections circulating in South Korea in recent years. G1 was the predominant genotype before 2000 and between 2004 and 2009; G2 and G3 were more prevalent during the seasons of 2000–2001 and 2003–2005, respectively; and G4 was predominant during the seasons of 2002–2003 and 2007–2008 [42]. G3 rotavirus genotypes have a broad host range and they have been discovered in several host species. G3 has been shown to be the dominant genotype and has spread throughout many Asian countries, including Japan [43], China [44], Vietnam [45], and Hong Kong [46]. Recently, the incidences of the G2, G3, and G4 genotypes decreased, while the incidence of the G1 genotype increased, suggesting that the predominant genotypes of rotaviruses in South Korea can change rapidly within a short period of time [33].

The P[9] genotype is frequently detected in cats [47], and the first P[9] human rotavirus, the K8 strain with G1 specificity, was isolated from a 14-year-old boy in Hokkaido, Japan in 1977 [48]. Although P[9] genotypes have been detected sporadically in most of the rotavirus surveillance studies conducted in South Korea, there has been no significant emergence of P[9] genotypes in the Korean population. The relative global frequency of the P[9] genotypes represents less than 2.5% of total rotavirus infections, and the majority of these genotypes carry G1, G2, and G3 specificity, except for a few isolates that have been found to have G4, G6, G9, and G12 specificity [20]. The present analysis of VP4/P[9] genes revealed a genetic relationship between the CAU12-2-51 strain and the recently identified P[9] strains, demonstrating that this strain had the highest identity to the feline rotavirus strain BA222.

Recent reports established a rotavirus classification system in which each gene of the rotavirus is assigned a particular genotype based on designated nucleotide identity cutoff values. Full genome

Table 1. Genotypes of the 11 segments of the CAU12-2-51 rotavirus strain with reference to strains available in the GenBank database.

Strain	Genotype										
	**VP7**	**VP4**	**VP6**	**VP1**	**VP2**	**VP3**	**NSP1**	**NSP2**	**NSP3**	**NSP4**	**SNP5**
RVA/Human-tc/KOR/**CAU12-2-51**/2012/G3P[9]	G3	P[9]	I2	R2	C2	M2	A3	N2	T3	E3	H3
RVA/Human-wt/ITA/**PAI58**/1996/G3P[9]	G3	P[9]	I2	R2	C2	M2	A3	N2	T6	E2	H3
RVA/Human-tc/ITA/**PAH136**/1996/G3P[9]	G3	P[9]	I2	R2	C2	M2	A3	N1	T6	E2	H3
RVA/Cat-wt/AUS/**BA222**/2005/G3P[9]	G3	P[9]	I2	R2	C2	M2	A3	N1	T3	E2	H3
RVA/Human-tc/JPN/**AU-1**/1982/G3P3[9]	G3	P[9]	I3	R3	C3	M3	A3	N3	T3	E3	H3
RVA/Human-tc/CHN/**L62**/2006/G3P[9]	G3	P[9]	I3	R3	C3	M3	A3	N3	T3	E3	H6
RVA/Human-wt/CHN/**E2451**/2011/G3P[9]	G3	P[9]	I3	R3	C3	M3	A3	N3	T3	E3	H6
RVA/Human-tc/THA/**CU-365**/2008/G3P[9]	G3	P[9]	I3	R3	C3	M3	A3	N3	T3	E3	H6
RVA/Cat-tc/AUS/**Cat2**/1984/G3P[9]	G3	P[9]	I3	R3	C2	M3	A3	N1	T6	E3	H3
RVA/Human-wt/JPN/**KF17**/2009-2010/G6P[9]	G6	P[9]	I2	R2	C2	M2	A3	N2	T3	E3	H3
RVA/Human-wt/HUN/**Hun5**/1997/G6P[14]	G6	P[14]	I2	R2	C2	M2	A11	N2	T6	E2	H3
RVA/Human-tc/USA/**DS-1**/1976/G2P1B[4]	G2	P[4]	I2	R2	C2	M2	A2	N2	T2	E2	H2
RVA/Human-tc/USA/**Wa**/1974/G1P1AP[8]	G1	P[8]	I1	R1	C1	M1	A1	N1	T1	E1	H1

Wa (G1P[8]), DS-1 (G2P[4]), and AU-1(G3P[9]) strains were used as references for genotype 1 (Wa-like), genotype 2 (DS-1 like), and genotype 3 (AU-1 like), respectively.
doi:10.1371/journal.pone.0097127.t001

Table 2. Nucleotide sequence identity of the 11 segments of the CAU12-2-51 rotavirus strain relative to some genetically related strains.

Gene	Cutoff value (%)*	Genotype of the CAU12-2-51 strain	Closely related strains and sequence identity (aa) (%) to CAU12-2-51		Sequence identity (aa) (%) of CAU12-2-51 with other related strains				
					AU-1/Hu (G3P[9])	DS-1/Hu (G2P[4])	CU-365-KK/Hu (G3P[9])	BA222/Fe (G3P[9])	KF17/Hu G6P[9]
VP7	83	G3	CU365-KK/Hu	96.0 (97.5)	90.2(94.1)	G2	96.0(97.5)	93.8 (96.0)	G6
VP4	84	P[9]	BA222/Fe	97.9 (99.2)	95.4(97.3)	P[4]	95.0(98.3)	97.9 (99.2)	97.8 (98.8)
VP6	85	I2	RUS-Nov-K2/Hu	98.5 (99.7)	I3	86.2(98.4)	I3	93.4 (99.7)	98.2 (99.7)
VP1	83	R2	Hun5/Hu	97.4 (99.1)	R3	86.3(97.4)	R3	84.9 (97.0)	93.8 (98.8)
VP2	84	C2	Hun5/Hu	97.2 (99.4)	C3	84.8(96.9)	C3	91.0 (98.9)	88.2 (99.2)
VP3	81	M2	KF17/Hu	97.8 (97.2)	M3	83.6(90.9)	M3	97.1 (98.0)	97.8 (98.8)
NSP1	79	A3	KF17/Hu	97.2 (96.7)	93.1(93.1)	A2	93.4 (94)	97.0 (96.5)	97.2 (96.7)
NSP2	85	N2	O211/Hu	99.4 (99.6)	N3	86.2(93.6)	N3	N1	98.9 (99.6)
NSP3	85	T3	AU-1/Hu	96.9 (97.7)	96.9(97.7)	T2	85.7 (95.5)	94.9 (97.4)	95.0 (97.7)
NSP4	85	E3	KF17/Hu	97.7 (97.1)	96.2(97.1)	E2	89.9 (92.5)	E2	97.7 (97.1)
NSP5	91	H3	KF17/Hu	98.8 (97.9)	93.1(92.9)	H2	H6	98.6 (97.4)	98.8 (97.9)

*Percentage nucleotide cutoff values and genotype proposed by Matthijnessens *et al*. (2008). Hu, Human; Fe Feline.
doi:10.1371/journal.pone.0097127.t002

analysis also provided an excellent platform for determining interspecies evolutionary relationships. To date, complete genome characterizations have assigned the G3P[9] strains to the genotype constellations G3-P[9]-I3-R3-C3-M3-A3-N3-T3-E3-H3 [12], G3-P[9]-I2-R2-C2-M2-A3-N1-T6-E2-H3 [36], G3-P[9]-I2-R2-C2-M2-A3-N2-T6-E2-H3 [36], G3-P[9]-I3-R3-C3-M3-A3-N3-T3-E3-H6 [7,21], G3-P[9]-I2-R2-C2-M2-A3-N1-T3-E2-H3 [9], G3-P[9]-I3-R3-C2-M3-A3-N1-T6-E3-H3 [18], and G3-P[9]-I2-R2-C2-M2-A3-N2-T1-E2-H3 [49]. The genotype of the human CAU12-2-51 strain exhibited a new G3-P[9]-I2-R2-C2-M2-A3-N2-T3-H3 genotype constellation.

Elucidation of the complete genomic sequence of the CAU12-2-51 strain revealed that the genes encoding VP7, NSP1, NSP3, NSP4, and NSP5 were related to the human AU-1-like genotype, together with the related human/feline-like and feline rotavirus strains. The remaining five genes encoding VP6, VP1–VP3, and NSP2 were related to the human DS-1-like genotype and the related human/bovine-like and bovine rotavirus strains. These results were in agreement with the hypothesis that genotypes of human rotavirus strains belonging to the AU-1-like and DS-1-like genogroups had a close evolutionary relationship with feline/canine and bovine rotavirus strains, respectively [12]. However, it is currently difficult to determine the specific origins of some of these genotypes because of the unavailability of sequence data for animal rotavirus strains, including feline and bovine rotaviruses. Therefore, additional sequence data for these strains are needed to understand the true origins of these viruses [12].

There are two approved vaccines for rotavirus, which were developed based on different strategies. Rotarix (GlaxoSmithKline Biologicals, Rixensart, Belgium) is a single, live-attenuated human G1P[8] rotavirus strain [50], while RotaTeq (Merck and Co. Inc., West Point, PA) is a pentavalent, live bovine-human reassortant vaccine containing the G1, G2, G3, G4, and P[8] genotypes [51]. RotaTeq and Rotarix were introduced in South Korea in September 2007 and July 2008, respectively. The patient in this study had not received any rotavirus vaccines and showed severe symptom of diarrhea. Comparison of the aa sequence of the *VP4* gene of vaccines and CAU12-2-51 strains revealed low sequence identity (34.2%–34.9%). Novel rotaviruses may contain aa changes in regions that have known biological functions, and therefore, we expect that the effectiveness of vaccines raised against these regions may be poor when attempting to prevent infection of such reassortment rotavirus strains [52].

In conclusion, the repeated detection of G3P[9] strains in many surveillance studies suggests that these strains may represent important strains currently circulating in South Korea. Whole genome sequencing of all 11 segments of the CAU12-2-51 strain revealed a unique pattern of genetic diversity. Phylogenetic analysis of the CAU12-2-51 strain indicated that this strain had a complex evolutionary origin, potentially involving reassortment events between feline and bovine rotaviruses. The *VP7*, *VP4*, *NSP1*, *NSP3*, *NSP4*, and *NSP5* genes were related to human/feline-like and feline rotavirus strains, and the remaining five genes, i.e., *VP6*, *VP1*, *VP2*, *VP3*, and *NSP2*, were the most related to human/bovine-like and bovine rotavirus strains. Taken together, our current data enhance our understanding of the diversity of rotavirus through molecular, genetic, evolutionary, and epidemiological analyses.

Supporting Information

Table S1 Primers used to amplify the VP7, VP4, VP6, VP1, VP2, VP3, NSP1, NSP2, NSP3, NSP4 and NSP5 gene segments described in this study.
(DOC)

Table S2 Related origin of the CAU12-2-51 rotavirus strain to reference strains used in this study.
(DOC)

Author Contributions

Conceived and designed the experiments: WK. Performed the experiments: SJ. Analyzed the data: VTT WK. Contributed reagents/materials/analysis tools: IL WK. Wrote the paper: SJ WK.

References

1. Tate JE, Burton AH, Boschi-Pinto C, Steele AD, Duque J, et al. (2012) 2008 estimate of worldwide rotavirus-associated mortality in children younger than 5 years before the introduction of universal rotavirus vaccination programmes: a systematic review and meta-analysis. Lancet Infect Dis 12: 136–141.
2. Matthijnssens J, Otto PH, Ciarlet M, Desselberger U, Van Ranst M, et al. (2012) VP6-sequence-based cutoff values as a criterion for rotavirus species demarcation. Arch Virol 157: 1177–1182.
3. Hu L, Crawford SE, Hyser JM, Estes MK, Prasad BV (2012) Rotavirus non-structural proteins: structure and function. Curr Opin Virol 2: 380–388.
4. Hoshino Y, Kapikian AZ (2000) Rotavirus serotypes: classification and importance in epidemiology, immunity, and vaccine development. J Health Popul Nutr 18: 5–14.
5. Matthijnssens J, Ciarlet M, McDonald SM, Attoui H, Banyai K, et al. (2011) Uniformity of rotavirus strain nomenclature proposed by the Rotavirus Classification Working Group (RCWG). Arch Virol 156: 1397–1413.
6. Trojnar E, Sachsenroder J, Twardziok S, Reetz J, Otto PH, et al. (2013) Identification of an avian group A rotavirus containing a novel VP4 gene with a close relationship to those of mammalian rotaviruses. J Gen Virol 94: 136–142.
7. Santos N, Hoshino Y (2005) Global distribution of rotavirus serotypes/genotypes and its implication for the development and implementation of an effective rotavirus vaccine. Rev Med Virol 15: 29–56.
8. Gentsch JR, Laird AR, Bielfelt B, Griffin DD, Banyai K, et al. (2005) Serotype diversity and reassortment between human and animal rotavirus strains: implications for rotavirus vaccine programs. J Infect Dis 192 Suppl 1:S146–159.
9. Banyai K, Laszlo B, Duque J, Steele AD, Nelson EA, et al. (2012) Systematic review of regional and temporal trends in global rotavirus strain diversity in the pre rotavirus vaccine era: insights for understanding the impact of rotavirus vaccination programs. Vaccine 30 Suppl 1:A122–130.
10. Matthijnssens J, Heylen E, Zeller M, Rahman M, Lemey P, et al. (2010) Phylodynamic analyses of rotavirus genotypes G9 and G12 underscore their potential for swift global spread. Mol Biol Evol 27: 2431–2436.
11. Wang YH, Pang BB, Zhou X, Ghosh S, Tang WF, et al. (2013) Complex evolutionary patterns of two rare human G3P[9] rotavirus strains possessing a feline/canine-like H6 genotype on an AU-1-like genotype constellation. Infect Genet Evol 16: 103–112.
12. Matthijnssens J, Ciarlet M, Heiman E, Arijs I, Delbeke T, et al. (2008) Full genome-based classification of rotaviruses reveals a common origin between human Wa-Like and porcine rotavirus strains and human DS-1-like and bovine rotavirus strains. J Virol 82: 3204–3219.
13. Nakagomi O, Nakagomi T, Akatani K, Ikegami N (1989) Identification of rotavirus genogroups by RNA-RNA hybridization. Mol Cell Probes 3: 251–261.
14. Birch CJ, Heath RL, Marshall JA, Liu S, Gust ID (1985) Isolation of feline rotaviruses and their relationship to human and simian isolates by electropherotype and serotype. J Gen Virol 66: 2731–2735.
15. Marshall JA, Kennett ML, Rodger SM, Studdert MJ, Thompson WL, et al. (1987) Virus and virus-like particles in the faeces of cats with and without diarrhoea. Aust Vet J 64: 100–105.
16. Mochizuki M, Nakagomi T, Nakagomi O (1997) Isolation from diarrheal and asymptomatic kittens of three rotavirus strains that belong to the AU-1 genogroup of human rotaviruses. J Clin Microbiol 35: 1272–1275.
17. Horie Y, Masamune O, Nakagomi O (1997) Three major alleles of rotavirus NSP4 proteins identified by sequence analysis. J Gen Virol 78: 2341–2346.
18. Tsugawa T, Hoshino Y (2008) Whole genome sequence and phylogenetic analyses reveal human rotavirus G3P[3] strains Ro1845 and HCR3A are examples of direct virion transmission of canine/feline rotaviruses to humans. Virology 380: 344–353.
19. Martella V, Potgieter AC, Lorusso E, De Grazia S, Giammanco GM, et al. (2011) A feline rotavirus G3P[9] carries traces of multiple reassortment events and resembles rare human G3P[9] rotaviruses. J Gen Virol 92: 1214–1221.
20. Khamrin P, Maneekarn N, Peerakome S, Tonusin S, Phan TG, et al. (2007) Molecular characterization of rare G3P[9] rotavirus strains isolated from children hospitalized with acute gastroenteritis. J Med Virol 79: 843–851.

21. Theamboonlers A, Maiklang O, Thongmee T, Chieochansin T, Vuthitanachot V, et al. (2013) Complete genome analysis of a rare human G3P[9] rotavirus posing as an AU-1 like strain. SpringerPlus 2: 569.
22. Than VT, Kim W (2013) Prevalence of rotavirus genotypes in South Korea in 1989-2009: implications for a nationwide rotavirus vaccine program. Korean J Pediatr 56: 465–473.
23. Seo JK, Sim JG (2000) Overview of rotavirus infections in Korea. Pediatr Int 42: 406–410.
24. Oh SA, Park SH, Ham HJ, Seung HJ, Jang JI, et al. (2013) Molecular Characterization of Norovirus and Rotavirus in Outbreak of Acute Gastroenteritis in Seoul. J Bacteriol Virol 43: 307–316.
25. Than VT, Park JH, Chung IS, Kim JB, Kim W (2013) Whole-genome sequence analysis of a Korean G11P[25] rotavirus strain identifies several porcine-human reassortant events. Arch Virol 158: 2385–2393.
26. Huh JW, Kim WH, Yoon MH, Lim YH (2009) Genotypic distribution of rotavirus strains causing severe gastroenteritis in Gyeonggi province, South Korea, from 2003 to 2005. Arch Virol 154: 167–170.
27. Matthijnssens J, Ciarlet M, Rahman M, Attoui H, Banyai K, et al. (2008) Recommendations for the classification of group A rotaviruses using all 11 genomic RNA segments. Arch Virol 153: 1621–1629.
28. Thompson JD, Gibson TJ, Plewniak F, Jeanmougin F, Higgins DG (1997) The CLUSTAL_X windows interface: flexible strategies for multiple sequence alignment aided by quality analysis tools. Nucleic Acids 25: 4876–4882.
29. Felsenstein J (1993) PHYLIP: phylogenetic inference package, version 3.5c.
30. Gascuel O, Steel M (2006) Neighbor-joining revealed. Mol Biol Evol 23: 1997–2000.
31. Saitou N, Nei M (1987) The neighbor-joining method: a new method for reconstructing phylogenetic trees. Mol Biol Evol 4: 406–425.
32. Jukes TH, Cantor CR (1969) Evolution of protein molecules. In Munro HN, editor, Mammalian Protein Metabolism, pp. 21–132, Academic Press, New York.
33. Matthijnssens J, Potgieter CA, Ciarlet M, Parreno V, Martella V, et al. (2009) Are human P[14] rotavirus strains the result of interspecies transmissions from sheep or other ungulates that belong to the mammalian order Artiodactyla? J Virol 83: 2917–2929.
34. Banyai K, Bogdan A, Domonkos G, Kisfali P, Molnar P, et al. (2009) Genetic diversity and zoonotic potential of human rotavirus strains, 2003–2006, Hungary. J Med Virol 81: 362–370.
35. Yamamoto D, Kawaguchiya M, Ghosh S, Ichikawa M, Numazaki K, et al. (2011) Detection and full genomic analysis of G6P[9] human rotavirus in Japan. Virus genes 43: 215–223.
36. De Grazia S, Giammanco GM, Potgieter CA, Matthijnssens J, Banyai K, et al. (2010) Unusual assortment of segments in 2 rare human rotavirus genomes. Emerg Infect Dis 16: 859–862.
37. Rahman M, Matthijnssens J, Yang X, Delbeke T, Arijs I, et al. (2007) Evolutionary history and global spread of the emerging g12 human rotaviruses. J Virol 81: 2382–2390.
38. Cohen J, Lefevre F, Estes MK, Bremont M (1984) Cloning of bovine rotavirus (RF strain): nucleotide sequence of the gene coding for the major capsid protein. Virology 138: 178–182.
39. Matthijnssens J, Rahman M, Van Ranst M (2008) Two out of the 11 genes of an unusual human G6P[6] rotavirus isolate are of bovine origin. J Gen Virol 89: 2630–2635.
40. Ghosh S, Shintani T, Urushibara N, Taniguchi K, Kobayashi N (2012) Whole-genomic analysis of a human G1P[9] rotavirus strain reveals intergenogroup-reassortment events. J Gen Virol 93: 1700–1705.
41. Maestri RP, Kaiano JH, Neri DL, Soares Lda S, Guerra Sde F, et al. (2012) Phylogenetic analysis of probable non-human genes of group A rotaviruses isolated from children with acute gastroenteritis in Belem, Brazil. J Med Virol 84: 1993–2002.
42. Ndze VN, Papp H, Achidi EA, Gonsu KH, Laszlo B, et al. (2013) One year survey of human rotavirus strains suggests the emergence of genotype G12 in Cameroon. J Med Virol 85: 1485–1490.
43. Thongprachum A, Chan-it W, Khamrin P, Okitsu S, Nishimura S, et al. (2013) Reemergence of new variant G3 rotavirus in Japanese pediatric patients, 2009–2011. Infect Genet Evol 13: 168–174.
44. Wang YH, Kobayashi N, Zhou X, Nagashima S, Zhu ZR, et al. (2009) Phylogenetic analysis of rotaviruses with predominant G3 and emerging G9 genotypes from adults and children in Wuhan, China. J Med Virol 81: 382–389.
45. Ngo TC, Nguyen BM, Dang DA, Nguyen HT, Nguyen TT, et al. (2009) Molecular epidemiology of rotavirus diarrhoea among children in Haiphong, Vietnam: the emergence of G3 rotavirus. Vaccine 27 Suppl 5:F75–80.
46. Mitui MT, Chan PK, Nelson EA, Leung TF, Nishizono A, et al. (2011) Co-dominance of G1 and emerging G3 rotaviruses in Hong Kong: a three-year surveillance in three major hospitals. J Clin Virol 50: 325–333.
47. Kapikian AZ, Hoshino Y, Chanock RM (2007) Rotaviruses. In: Knipe DM, Howley PM, Griffin DE, Martin MA. editors. Fields virology. 5th ed. Philadelphia: Lippincott Williams & Wilkins 1787–825.
48. Taniguchi K, Nishikawa K, Urasawa T, Urasawa S, Midthun K, et al. (1989) Complete nucleotide sequence of the gene encoding VP4 of a human rotavirus (strain K8) which has unique VP4 neutralization epitopes. J Virol 63: 4101–4106.
49. Grant L, Esona M, Gentsch J, Watt J, Reid R, et al. (2011) Detection of G3P[3] and G3P[9] rotavirus strains in American Indian children with evidence of gene reassortment between human and animal rotaviruses. J Med Virol 83: 1288–1299.
50. Ward RL, Bernstein DI (2009) Rotarix: a rotavirus vaccine for the world. Clin Infect Dis 48: 222–228.
51. Heaton PM, Ciarlet M (2007) Vaccines: the pentavalent rotavirus vaccine: discovery to licensure and beyond. Clin Infect Dis 45: 1618–1624.
52. Donato CM, Cannan D, Bogdanovic-Sakran N, Snelling TL, Kirkwood CD (2012) Characterisation of a G9P[8] rotavirus strain identified during a gastroenteritis outbreak in Alice Springs, Australia post Rotarix vaccine introduction. Vaccine 30 Suppl 1:A152–158.
